# Juvenile Nasopharyngeal Angiofibroma: Magnetic Resonance Imaging Findings

**DOI:** 10.5334/jbr-btr.1090

**Published:** 2016-06-01

**Authors:** Ayse Gul Alimli, Murat Ucar, Cigdem Oztunali, Koray Akkan, Oznur Boyunaga, Cagrı Damar, Betül Derinkuyu, Nil Tokgöz

**Affiliations:** 1 Gazi University School of Medicine, TR

**Keywords:** Juvenile nasopharyngeal angiofibroma, Magnetic Resonance Imaging, spread pattern, nasopharyngeal mass, angiography

## Abstract

**Purpose:**

Juvenile nasopharyngeal angiofibroma (JNA) is a rare tumor that exhibits a predictable spreading pattern. Radiologist’s prior knowledge on the tumor’s characteristics aids in establishing a diagnosis. We aimed to report the characteristic Magnetic Resonance Imaging (MRI) findings and the spread patterns of JNA.

**Materials and methods:**

We retrospectively evaluated the MRI findings and extension pathways of 6 cases of JNA.

**Results:**

The patients’ age ranged from 8 to 16 years and all patients were male. The tumors were classified according to the Onerci system. Tumors were largely isointense to muscle on T1-weighted images and hyperintense on T2-weighted images. All lesions had internal signal-void regions and all exhibited intense enhancement after IV contrast injection. Diffusion restriction was not an associated feature. ADC values for these tumors were high. The evaluation of the available MR angiography studies of three patients showed the blood supply to the tumor to be mainly from the internal maxillary branch of the external carotid artery. In all patients, the diagnosis was based on MR images and a surgical excision was planned.

**Conclusion:**

The diagnosis can be established based on the characteristic imaging findings and the clinical history without performing a biopsy.

## Introduction

Juvenile nasopharyngeal angiofibroma (JNA) is a rare tumor that predominantly occurs in adolescent males, the average age of occurence being 15 [1]. It is the most common benign tumor of the nasopharnyx but only accounts for less than 0.05 per cent of all head and neck tumors [2]. Although a histopathologically benign tumor of vascular origin, JNA behaves locally invasive and exhibits an aggressive clinical course [3,4]. Erosion of the adjacent anatomical structures, particularly of the bones, may result in intracranial extension of this tumor.

Many theories regarding the pathogenesis of JNA have been proposed. Given the high prevalance of JNA among adolescent boys, one theory suggests that the tumor may be hormone dependent. Although the genetic studies have shown the expression of estrogen-androgen receptors by JNAs, an association with increased serum hormone levels hasn’t been proved, and the influence of the hormones on these tumors remains debatable [1,5].

A review of the literature reveals rare cases of female and older male patients with JNA [6,7,8,9]. Malignant transformation of the tumor is also a rare finding and reported to be associated with recurrent radiotherapy [10].

JNA is thought to originate from the posterior-superior part of the sphenoplatine foramen, which is located on the posterolateral wall of the nasal cavity. The tumor is located in close relationship with the pterygoplatine fossa, and although growing slowly, it may extend into the infratemporal fossa, the maxillary sinus, nasal cavity, orbita, sphenoid sinus, skull base, and into the cranial cavity [3,11].

The typical symptoms of JNA are unilateral progressive nasal obstruction (80%–90%) and recurrent epistaxis (45%–60% ) [12]. Recognition of the characteristic imaging findings of this tumor, along with the clinical history and physical examination findings of the patients, is important for the diagnosis. Computed tomography (CT) and magnetic resonance imaging (MRI) are the two preferred modalities that are used to detect and to determine the extent of these tumors [1]. In most cases, a diagnosis of JNA can be established based on the characteristic imaging findings, without performing a biopsy [13].

The gold-standard treatment of JNA is the surgical excision of the tumor after a preoperative embolization procedure. Due to the complex anatomy of the skull base, excision of the advanced tumors may be challenging, and chemotherapy, radiotherapy, or hormone therapy may be added to the treatment.

The recurrence risk of the tumor is largely determined by the extent of the tumor at the time of diagnosis and by the success of the subsequent surgery. Accurate delineation of the location and the extent of the tumor is crucial for the preoperative planning. The radiologist’s prior knowledge of the tumor’s MRI characteristics and its extranasopharyngeal spread pathways through the complex skull base structures aids in establishing a diagnosis and guides the surgeon [1].

In this study, we aimed to report the characteristic MRI findings and the spread patterns of this rare tumor. In addition, we purpose to emphasize that the diagnosis of JNA can be established based on the characteristic imaging findings, without performing a biopsy. A search of our archive from 2008 to 2014 revealed six JNA cases, and we retrospectively evaluated the MRI findings of these tumors and their extension pathways.

## Material and Methods

### Patients

The medical records of patients who were operated on for a tumor of the sinonasal cavity or nasopharynx in the ear, nose, and throat clinic of our hospital between 2008 and 2014 were reviewed. Of these, six patients who were histopathologically diagnosed with JNA and had their preoperative MR studies at our department were included in the study.

### MRI Protocol

Magnetic resonance imagings were performed on 1,5-T (Signa Excite; GE Healthcare, Milwaukee, Wis) and 3-T systems (Siemens Magnetom Verio, Erlangen, Germany). For all six patients, T1-weighted images in axial and sagittal planes, axial T2-weighted, and fat-saturated coronal T1-weighted images; coronal short tau inversion recovery (STIR) images; and fat-saturated contrast-enhanced T1-weighted images in axial, coronal, and sagittal planes were included in the imaging protocol. Four patients had their additional diffusion-weighted images and ADC maps. Two patients were evaluated with time-of-flight (TOF) MR angiography of their carotid arteries, and a TWIST-MR angiography (time-resolved angiography with stochastic trajectories) was available for review in one patient.

### Image Analysis

MR images were evaluated by two experienced radiologists in a concensus reading. The lesions’ primary location and extent, contour and signal-intensity characteristics, contrast-enhancement patterns, and feeding arteries were analysed, and additional lesion-associated findings were noted if present. In four patients, diffusion-weighted images were evaluated, and the lesions’ mean ADC values were measured. All lesions were staged according to Radkowski and Onerci staging systems. For all patients, the clinical history and the physical examination findings were available from the medical records.

## Results

The patients’ ages ranged from 8 to 16 years (mean age, 12,5 years), and all patients were male. The primary presenting symptom was nasal obstruction in three patients and epistaxis in two patients; one patient had combined nasal obstruction and epistaxis. The tumor was primarly located on the right side in five patients and on the left side in one patient. All tumors had lobulated contours.

Tumor extension into the nasopharynx, the nasal cavity, and the pterygopalatine fossa was a common feature in all patients. The infratemporal fossa was involved in five cases, the maxillary sinuses were involved in two, and the sphenoid sinus and the ethmoid air cells were involved in four cases. The cheek region was involved in two patients, and two patients had orbital involvement. The tumor extended into the cavernous sinus in three patients. The Holman-Miller sign was present in three cases. Erosion of the adjacent bones was found in four patients, and all patients demonstrated medullary bone marrow edema. Of the six cases, intratumoral cystic components were observed in four.

**Figure 1:**
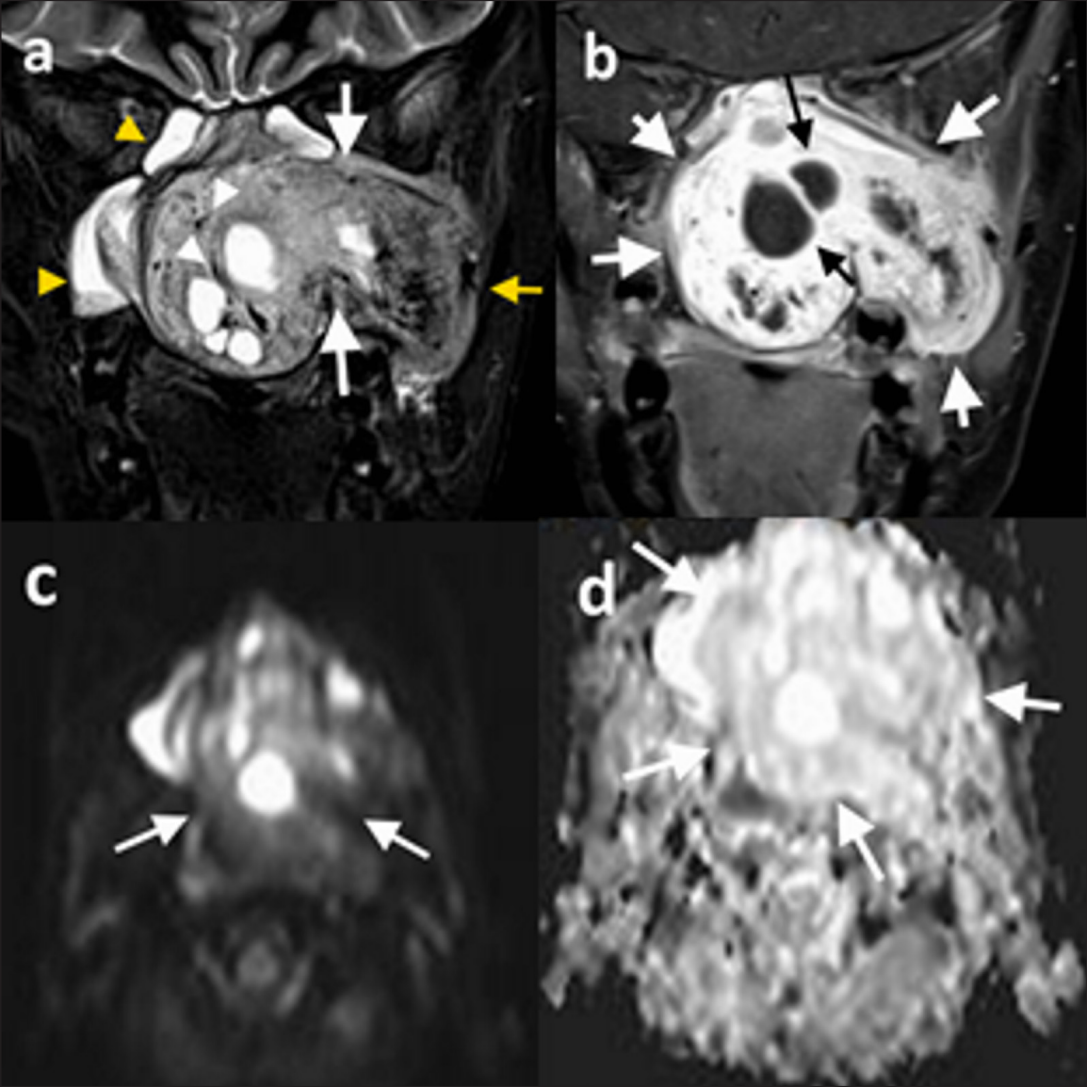
13-year-old male patient (patient 5), **(a)** coronal T2-weighted precontrast, **(b)** coronal T1-weighted postcontrast, **(c)** DWI, and **(d)** ADC map demonstrate a left-sided nasopharyngeal mass which enlarges the ipsilateral pterygopalatine fossa (*a, white arrows*) and extends into the temporal fossa (*a, yellow arrow*). The mass is hyperintense on T2-weighted image (a) and exhibits significant contrast enhancement (b). Diffusion-weighted images (c) showed no diffusion restriction, and the lesion has high signal intensity on the ADC map (d). The tumor demonstrates internal cystic components (*b, black arrows*) and signal-void regions (*a, white arrowheads*). Image a demonstrates inflammatory signal changes in maxillary and sphenoid sinuses (*yellow arrowheads*).

MRI signal intensity of the tumors were heterogeneous in all cases. Tumors were largely isointense to muscle on T1-weighted images and hyperintense on T2-weighted images. All lesions had internal signal-void regions, and all exhibited intense enhancement after intravenous (IV) contrast injection. In four patients for whom the diffusion-weighted images were available, diffusion restriction was not an associated feature (Figure 1). ADC values for these tumors were high, with a mean value of 1.6 ×10^–3^ mm^2^/s, consistent with benign lesions.

**Figure 2:**
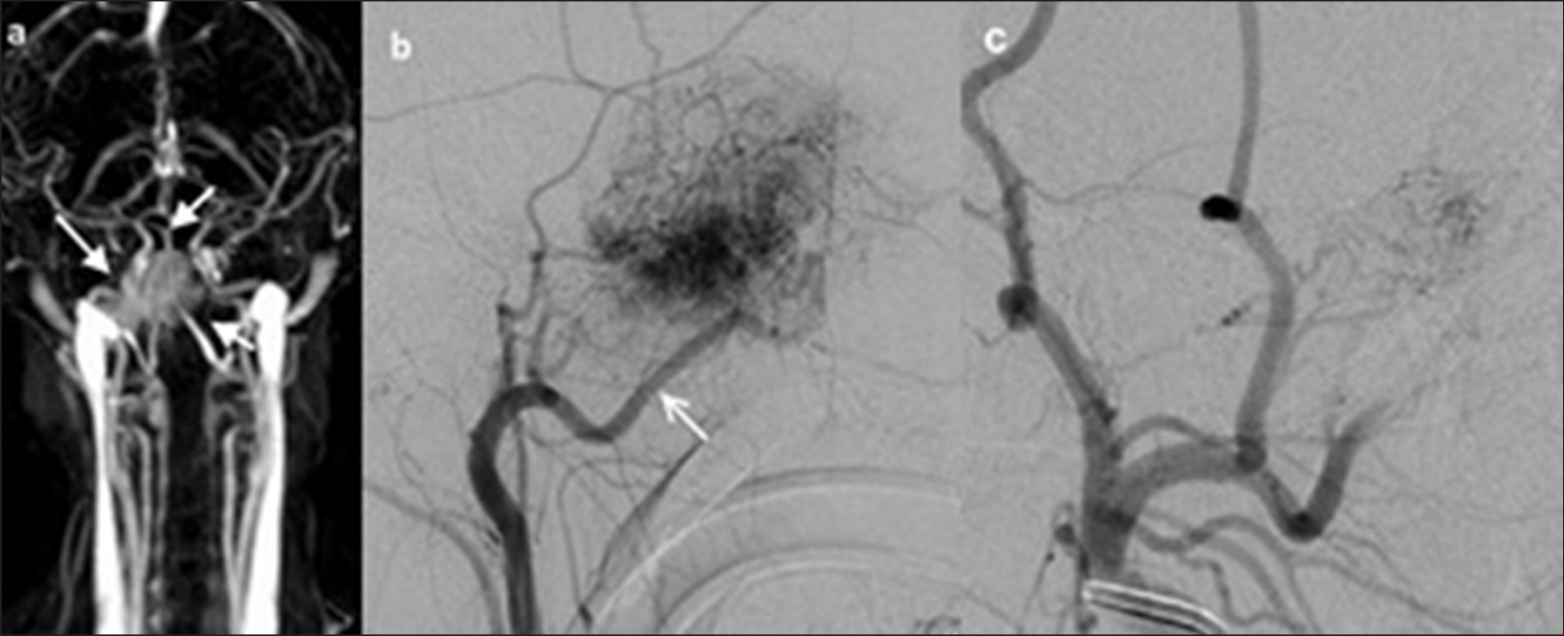
10-year-old male patient (patient 3), **(a)** TWIST-MR angiography of the patient showed bilobule hypervasculer mass on the right side (*white arrows*). **(b)** Selective right carotid artery angiography shows that JNA is supplied with the right internal maxillary artery (*white arrow*). **(c)** It is observed that the opacification of JNA mainly dissappeared in the angiography display obtained after the internal maxillary artery was embolized with microcoil.

The evaluation of the available carotid TOF MR angiography studies of two patients and the TWIST-MR angiography of one patient showed hypervasculer masses (Figure 2) and the blood supply to the tumor to be mainly from the internal maxillary branch of the external carotid artery. In these angiographic studies, tumors didn’t seem to exhibit mass effect on adjacent vascular structures.

Due to mass effect of the tumors, inflammatory signal changes in mastoid air cells were observed in three patients and also in the paranasal sinuses of five patients. The perioptic cerebrospinal fluid space was enlarged in one patient, and one case had a mucous-retention cyst of the maxillary sinus.

In all patients, the diagnosis was based on MR images, and a surgical excision was planned. To reduce the intraoperative blood loss and morbidity, five patients underwent a preoperative selective embolization procedure. In one case (patient 5), the embolization wasn’t performed. The tumors were supplied with the internal maxillary artery in three patients, sphenopalatine artery in one patient, and external and internal carotid systems in one patient. Magnetic resonance imaging findings of the patients are listed in Table 1, and extension areas of the tumors are listed in Table 2.

**Table 1:** Magnetic Resonance İmaging Findings of the Patients.

Patient No	1	2	3	4	5	6

Tumor size	43 × 40 × 47	72 × 70 × 64	52 × 62 × 45	38 × 43 × 33	101 × 72 × 81	54 × 58 × 48
Contrast enhancement	Intense	Intense	Intense	Intense	Intense	Intense
Flow-voids	+	+	+	+	+	+
T1WI	Hyperintense	Isointense	Iso-/Hyperintense	Isointense	Isointense	Iso-/Hypointense
T2WI	Hyperintense	Iso-/Hyperintense	Hyperintense	Iso-/Hyperintense	Hyperintense	Iso-/Hyperintense
DWI	–	–	–	NA	–	NA
ADC value (mm2/s)	1,5 × 10–3	1,5 × 10–3	1,6 10–3	NA	1,7 × 10–3	NA
Antral sign	+	–	–	+	–	+
Cystic component	–	+	+	–	+	+
Vascular compression	–	–*	–	–	–	–

*NA* Not available. *Surrounds the internal carotid artery without compression.

**Table 2:** Extension Areas of the Tumors.

Patient no.	1	2	3	4	5	6

Localization	Right	Right	Right	Right	Left	Right
Nasopharynx	+	+	+	+	+	+
Nasal cavity	+	+	+	+	+	+
Pterygopalatine fossa	+	+	+	+	+	+
Infratemporal fossa	–	+	+	+	+	+
Maxillary sinus	–	+	–	–	+	–
Ethmoid sinus	–	+	+	–	+	+
Sphenoid sinus	–	+	+	–	+	+
Cheek	–	+	–	–	+	+
Orbit	–	+	–	–	+	–
Cavernous sinus	–	+	–	–	+	+
Optic chiasm	–	+	–	–	–	–
Skull base erosion	–	+	+	–	+	+
Middle cranial fossa	–	+	+ dural	–	+ dural	–
Bone marrow edema	+	+	+	+	+	+

Of the two early-stage cases (one was followed up for 2,5 years, and the other was followed up for 1 month), postoperative and follow-up MR studies were negative for any residual or recurrent lesion. One patient was lost to follow-up after the operation. Three patients that were initially diagnosed with advanced-stage lesions had residual lesions on postoperative MR studies. Of these three, one patient was followed up for a 7-month period; one for 2,5 years, and one for 3 years. All three patients with residual lesions received radiotherapy, and one of these was reoperated after a 1,5-year period for a growing residual tumor.

## Discussion

Juvenile nasopharyngeal angiofibroma is a locally aggressive, polypoid tumor of the sphenopalatine foramen that originates from the primitive mesenchyme. It is a highly vascular tumor and occurs in adolescent males. Due to its intense vascular supply, diagnostic biopsies are mostly avoided. Thus, the patient history and the clinical and imaging findings play an important role in diagnosis. In this study, the mean age of the patients and their chief complaints on admission were consistent with the literature. In all cases, the diagnosis of JNA was based on MRI findings, and the tumor extension was evaluated with MRI.

The gold-standard treatment of JNA is the surgical resection of the tumor, and surgical options include endoscopic surgery. Endoscopic surgery is advantageous for cosmetic reasons and also has the main advantage of less intraoperative blood loss. However, the use of the endoscopic surgical approach for locally invasive advanced-stage lesions is limited. Preoperative tumor embolizations performed with direct or transarterial injections of the liquid embolic agents are now frequently used to reduce the intraoperative blood loss. Radiotheraphy, alone or combined with surgery, may be used in the treatment of partially resectable or advanced-stage tumors. The reported recurrence rates for JNA are between 20 per cent and 50 per cent, and advanced-stage or large-sized lesions carry the risk of higher recurrence rates. For all the above mentioned reasons, recognition of the tumor’s imaging characteristics and its extension pathways is essential for the accurate preoperative staging of these tumors and determines the choice of treatment, including the postoperative radiotherapy [1,3].

**Figure 3:**
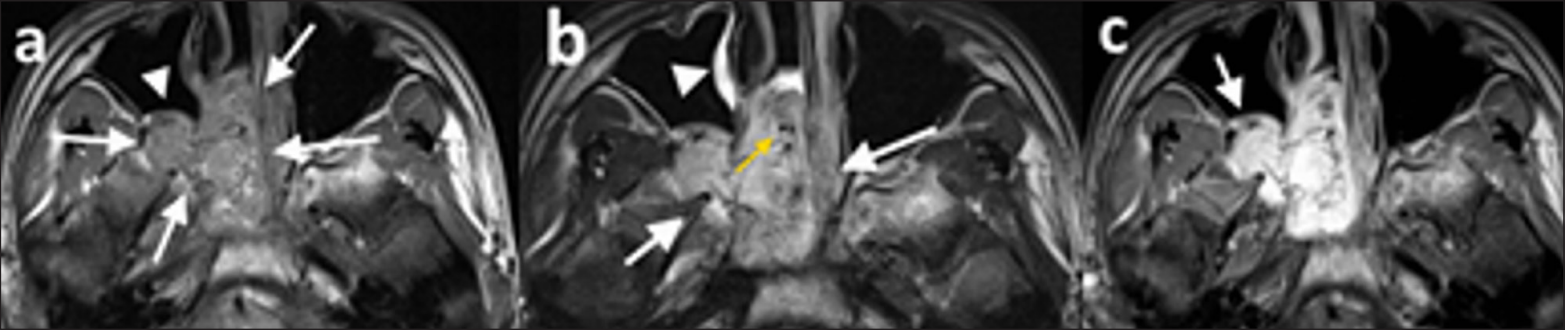
15-year-old male patient (patient 1), **(a)** axial T1-weighted precontrast, **(b)** axial T2-weighted precontrast, and **(c)** axial T1-weighted postcontrast MR images demonstrate a right-sided nasopharyngeal mass which enlarges the ipsilateral pterygopalatine fossa (*a and b white arrows*). The mass is isointense with the muscle on T1-weighted image (a) and hyperintense on T2-weighted image (b) and exhibits significant contrast enhancement (c). The posterior wall of the maxillary sinus demonstrates anterior bowing with resultant Holmann Miller sign (*a, white arrowhead; c, arrow*). The tumor demonstrates signal-void regions (*b, yellow arrow*) and inflammatory signal changes in maxillary sinuses (*b, white arrowhead*).

JNA is a histopathologically benign tumor, but it extends into adjacent foramina, fissures, and sinonasal spaces and demonstrates a locally invasive behaviour [1,14]. Although it may extend into unexpected locations, it usually follows a predictable spread pattern. Originating from the sphenopalatine foramen, the pterygoid process, and the sphenoid sinus, JNA mostly extends medially and laterally, largely because it encounters less resistant barriers in these directions [6]. Medially, the tumor usually invades the nasopharynx, the nasal cavity, and the maxillary and ethmoid sinuses. Laterally, it invades the pterygopalatine fossa and causes an anterior bowing of the posterior wall of the maxillary sinüs (Holmann-Miller sign) (Figure 3). Through the pterygomaxillary fissure, the tumor extends into the infratemporal fossa and laterally into the cheek [1,6].

Through the skull base, JNA may extend posteriorly into the pterygoid canal, pterygoid processes, and parapharyngeal space. It may erode the pterygoid processes and reach the greater wing of the sphenoid bone through the involvement of the medullary bone marrow. Also, from the infratemporal fossa, the tumor may extend into the orbita through the inferior orbital fissure. From the orbita, it may extend into the middle cranial fossa and the parasellar region through the superior fissure, where it may encase the internal carotid artery and invade the cavernous sinus. Invasion of the roof the sphenoid sinus and medial extension from the cavernous sinus may result in intracranial involvement as well as the invasion of the roof of the infratemporal fossa, foramen rotundum, or foramen ovale. Rarely, the tumor may reach the intracranial space through the invasion of the anterior ethmoid cells and the base of the anterior cranial fossa. The intracranial extension of the tumor is frequently limited to the extradural compartment [1,6,15,16].

The pterygopalatine fossa, nasopharynx, and nasal cavity were invaded in all patients in this study. Two cases predominantly exhibited a lateral extension pattern with the involvement of the infratemporal fossa. Three cases in the study had advanced-stage tumors and exhibited both lateral and medial extensions.

CT and MRI are the two primary imaging modalities used in the diagnosis and staging of these tumors. CT or MR angiography studies may also be used to detect the feeding vessels [13,16,17]. CT is superior in demonstrating the bone erosions and the invasion of the sphenoid bone, and MRI is more beneficial for the evaluation of the soft tissue, medullary bone marrow, and intracranial involvement [13]. MRI is the preferred modality for post-treatment follow-up, and recurrences are detected in approximately 25 per cent of the patients [9]. MRI is also helpful in differentiating the postoperative granulation tissue from a recurrent lesion.

On MR images, JNA demonstrates low signal-intensity on precontrast T1-weighted sequences and medium- to high-signal intensity on T2-weighted sequences [1]. However, most of the cases in our series showed isointensity to muscle on T1-weighted images, and one case exhibited high-signal intensity. On T2-weighted images, the tumors were of isohyperintense signal. Intratumoral signal voids and intense enhancement of the tumor after IV contrast injection are characteristic MRI features of JNA, and we observed these findings in all of our patients. None of the lesions in this study demonstrated diffusion restriction on DW images, and their mean ADC values were high, as expected in hypocellular benign tumors. The lesions’ lateral extensions were appreciated better from axial and coronal images, while the evaluation of the superior extensions of the tumors were easier using the sagittal and coronal images [1,13]. Although the presence of intratumoral degenerative cystic components is a rare radiological finding of JNA, we observed this feature in four out of six patients (Figure 1).

Medullary marrow edema of the sphenoid bone is a feature that is known to negatively affect the success of the surgery. Improved contrast resolution of the MRI and the use of fat-supressed sequences have facilitated the detection of the medullary bone marrow edema. Another advantage of MRI is that the fluid collections and the mucosal diseases of the paranasal sinuses are easily distunguished from the intrasinusoidal tumor components [6,14,15]. Inflammatory mucosal thickenings of the paranasal sinuses were readily distinguishable from the adjacent intrasinusoidal tumoral lesions in this study.

MRI plays an essential role in detecting the intracranial, dural, and intracavernosal extensions of the tumor and demonstrates the relation of the tumor with the internal carotid arteries and the pituitary gland. However, detecting the dural involvement may be challenging. Dural contrast enhancement on postcontrast T1-weighted images is an important sign of dural invasion. Also, contrast-enhanced FLAIR sequences are reported to be more sensitive in detecting leptomeningeal spread [18].

The expansion of the pterygopalatine fossa due to the presence of a nasopharygeal mass causes anterior bowing of the posterior wall of the maxillary sinus, and this sign (the antral sign or Holmann-Miller sign) is one of the characteristic findings of JNA [6,17]. Three out of six patients in this study exhibited Holmann-Miller sign, and the pterygopalatine fossa was expanded in all of our cases.

JNA is frequently supplied by the sphenopalatine and the maxillary arteries, especially in its early stage. Vascular supply from other external carotid artery branches or from the vertebral and internal carotid artery branches is also possible [11]. In this study, three patients were evaluated with additional MR angiography studies (two with TOF MR angiography and one with TWIST MR angiography), and the vascular supply to the tumor was from the external carotid artery branches in all three cases, as shown by digital subtraction angiography.

Staging systems for JNA is mostly based on the tumor location and extent. Various staging systems are described by different authors, including Radkowski [19], Fisch [20], Andrews [21], and Onerci [22]. The most commonly used staging systems are those described by Andrews (1989) and Radkowski (1996) [1]. In this study, we used Radkowski and Onerci staging systems. Table 3 summarizes the stage distribution of our patients, and Table 4 summarizes Radkowski and Onerci staging systems.

**Table 3:** Preoperative Stages of the Patients, According to Radkowski and Onerci Staging Systems.

Patient no.	Radkowski	Onerci

1	IIB	II
2	IIIB	IV
3	IIIA	III
4	IIC	II
5	IIIA	IV
6	IIIA	IV

**Table 4:** Summary of the Staging Systems.

Radkowski et al. 1996

IA Tumor limited to nasal cavity/nasopharynx IB Extension into one or more paranasal sinuses IIA Minimal extension into pterygopalatine fossa IIB Invasion of the pterygomaxillary fossa (with or without erosion of the orbital bones IIC Tumor extension into infratemporal fossa, with or without the involvement of the cheek or the pterygoid plates IIIA Erosion of the skull base, with minimal extension into cranial fossa IIIB Prominent intracranial extension, with or without invasion of the cavernous sinus
**Onerci et al. 2006**
I Minimal extension into nasal cavity, nasopharynx, ethmoid-sphenoid sinuses, or pterygomaxillary fossa II Extensive invasion of maxillary sinuses and the pterygomaxillary fossa, extension into anterior cranial fossa and limited extension into infratemporal fossa III Marrow involvement of the body and the greater wing of sphenoid bone and base of the pterygoid; extensive involvement of the infratemporal fossa, pterygoid plate, or orbital region; obliteration of cavernous sinus IV Intracranial extension between the pituitary and ICA, tumor located lateral to ICA, extension into middle cranial fossa

The recurrence risk is largely determined by the stage of the tumor and by the surgical approach that is used. Preoperative staging of the tumor is of paramount importance in surgical planning and directly affects the surgical approach and the success of the operation. Thus, the imaging studies are expected to provide accurate information regarding the extent of the lesion and to guide the surgeon.

JNA exhibits characteristic imaging findings and its spread pathways are largely predictable. MRI is the most commonly used modality for these tumors due to lack of the ionizing radiation, its multiplanar image acquisiton capability, increased soft tissue contrast resolution, its high sensitivity in demonstrating medullary marrow edema and the tumor vascularity, and its ability to differentiate the mucosal inflammation-fluid retentions from the tumoral invasion. MRI is also the preferred modality for the postoperative follow-up period. Recognition of the MRI characteristics of JNA and its extension pathways ensures an accurate staging of the tumor and directly affects the success of the treatment.

## Conclusion

Despite our retrospective study including a relatively low number of subjects, we concluded that MRI accurately delineates the extent of these tumors and aids in staging. The diagnosis can be established based on the characteristic imaging findings without performing a biopsy. The differential diagnosis includes nasopharyngeal carcinoma, lymphoma, rhabdomyosarcoma, sinonasal polyp, and adenoid hypertrophy. DWI and their mean ADC values can be used for distinguishing between benign and malignant tumors. In adolescent males, a nasopharygeal tumor that exhibits intense contrast enhancement with intratumoral signal voids and extends into the pterygopalatine fossa with the destruction of the pterygoid processes strongly supports the diagnosis of a JNA. Also, it may include cystic components.
